# Anterograde and Retrograde Effects of Benzodiazepines on Memory

**DOI:** 10.1100/tsw.2006.243

**Published:** 2006-11-16

**Authors:** Daniel Beracochea

**Affiliations:** Laboratoire Neurosciences Cognitives, UMR CNRS 5106 Univ. Bordeaux1, Bat Biologie Animale, Avenue des Facultés 33405 Talence-Cédex, France

**Keywords:** amnesia, benzodiazepines, memory, retrieval, implicit memory, explicit memory

## Abstract

Benzodiazepines are known as “acquisition-impairing” molecules, and their effects on anterograde memory processes are well described. In contrast, the impact of benzodiazepines on retrograde memory and, more particularly, on retrieval processes, is only marginally studied. This mini-review provides an overlook of the main studies evidencing an effect of benzodiazepines on retrograde memory, both in humans and animals, with special emphasis on retrieval processes. The conditions for the emergence of the benzodiazepine-induced retrieval impairments are also discussed.

## THE BENZODIAZEPINES

GABA ($\gamma$-amino butyric acid) is an amino acid that exherts a fast, inhibitory neurotransmission in the central nervous system. GABA receptors are of two kinds: GABA-A and GABA-B. GABA-A receptors are of a huge diversity and are functionally linked to benzodiazepine receptors. GABA-A receptors are pentameric membrane proteins that operate as GABA-gated $Cl^-$ channels. They are assembled from several families of subunits, of which at least 19 occur in the central nervous system. However, the vast majority of receptors appears to be associations of two $\alpha$-subunits, two ß-subunits, and a single $\gamma$-subunit, which comprise a central ion channel. The majority of them contain a benzodiazepine-binding site located at the interface of the $\gamma_2$-subunit and the respective $\alpha$-subunit ($\alpha_1$, $\alpha_2$, $\alpha_3$, or $\alpha_5$)[1]. Specifically, sedative and anterograde amnesic effects of benzodiazepines were mainly attributed to $\alpha_1$-containing GABA-A receptor subtypes, anxiolytic action to the $\alpha_2$-containing receptors, anticonvulsant activity partially, but not fully, to the $\alpha_1$-containing receptors, and muscle relaxant effect largely to the $\alpha_2$-containing receptors[2].

The benzodiazepine receptors themselves include “central” and “peripheral” types, only the central type being linked to GABA-A receptors. There are two main subtypes of GABA-A receptors: BZ-1 (preferentially located in the cerebellum) and BZ-2 (preferentially located in the hippocampus and the cortex). In so far as benzodiazepines exert their effects via the GABA-A receptors, most of the studies on the relationships between GABAergic activity and memory have been centered on the BZ-2 type, using benzodiazepines receptors ligands[3].

Three kinds of allosteric modulators act through the benzodiazepine-binding site: positive (agonist), neutral (antagonist), and negative (inverse agonist) modulators[4]. It is well established that agonists at the benzodiazepine site present anxiolytic and amnesic properties, whereas inverse agonists, such as β-carbolines, exert anxiogenic and learning-enhancing actions[5,6,7,8].

It is of importance to pinpoint that 1,4-benzodiazepines is a nonhomogenous chemical family, as regards the pharmacological spectrum and the half-lives of elimination. Thus, some of them are prescribed for their anxiolytic properties (such as diazepam, lorazepam, and oxazepam), others for their hypnotic properties (temazepam, flunitrazepam, or nitrazepam) or even for their anticonvulsant properties on epileptic seizure (diazepam)[9]. Moreover, half-life elimination varies from 1–4 h for triazolam to 32–47 h for diazepam.

In parallel, most of these compounds are also known for their effects on memory processes. Indeed, benzodiazepines also induced a severe anterograde amnesia[1,9,10,11].

## BENZODIAZEPINE EFFECTS ON MEMORY TYPES AND MEMORY STAGES

There are multiple ways to define memory: (1) duration (short-term and long-term spans), (2) contents (explicit and implicit processes), and (3) stages (acquisition, consolidation, or retrieval). Explicit memory belongs to the declarative memory system and involves the conscious recollection of past events (episodic memory). In contrast, implicit memory belongs to the nondeclarative system and involves a nonconscious (i.e., automatic or spontaneous) processing of information, as observed in the execution of procedural learning or in priming tasks. It has often been assumed that explicit memory is the only aspect of memory vulnerable to organic amnesia, given the involvement of the hippocampus in this type of memory[12,13]. If one takes into account these distinctions, it has been found that benzodiazepines have different effects on memory according to the duration, the types, or the stages of memory tested.

On the one hand, it is well known that benzodiazepine administration induced episodic memory deficits and that these impairments are mainly observed on long-term rather than on short-term memory[9]. On the other hand, it has been reported that benzodiazepines, such as diazepam, produced much more impairments in explicit memory tasks, whereas implicit ones would be spared[14,15]. Nevertheless, further studies have shown that the relative sensitivity of implicit memory to benzodiazepines depends on the compound used and of the time-dependent effects related to the relative state of absorption. Thus, on the one hand, recent studies have shown that the benzodiazepine lorazepam differs from other benzodiazepines in its impairing effects on implicit memory tasks[16,17]; on the other hand, it has been reported that oxazepam differently affected both implicit and explicit memory as a function of the time-course effects of that compound[18]. Nevertheless, the implicit memory deficit could be due to a possible contamination of the implicit memory task by an explicit retrieval memory strategy[19,20]. Using a process-dissociation procedure in a word-stem completion task that provides uncontaminated estimates of conscious and automatic memory processes, deficits of the automatic use of memory have been reported following lorazepam administration[21].

In addition to their effects on these two types of memory, benzodiazepines are mainly known as "acquisition-impairing" molecules. As it is well established, memory is composed of three stages: acquisition, consolidation, and retrieval. The evaluation of the effects of benzodiazepines on these different stages in animals depends on the occurrence of the drug administration: before the learning session (acquisition), immediately after (consolidation), or before the test session (retrieval). Nevertheless, one might be cautious in interpreting the data, since these experimental procedures did not fully isolate experimentally the different stages of memory, because experimental techniques may affect two or more stages depending on the time course of the manipulations[22]. Additionally, learning and memory can be affected by side effects of the compounds on motor function (sedation) or on other cognitive processes (attention, motivation, and arousal level)[23], as well as on emotion[24,25].

Several studies in human subjects have provided evidence that the impairment induced in memory processing by benzodiazepines clearly concerns the acquisition of information. Thus, the administration of benzodiazepines before the learning of a list of words or geometrical patterns impairs the recall of these lists in a later test; this impairment being a function of the dose administered[10,26]. Similar findings have also been found in animals in a wide variety of tasks and species[27,28,29,30,31]. In regard to the main data evidencing anterograde amnesia, benzodiazepines would impair acquisition processes mainly by disrupting the ability to build new associations between events.

## BENZODIAZEPINES AND RETRIEVAL PROCESSES

Interestingly, contrary to the anterograde impairment, most authors agree that benzodiazepines do not produce retrograde amnesia; that is to say, forgetting events memorized just before the administration of benzodiazepines[5,32,33]. It is, however, well evidenced that benzodiazepines act on other stages of memory[34]. Indeed, retrograde impairments in spatial memory tasks have been reported in animals, but mainly in pharmacological procedures in which benzodiazepines were administered just after the acquisition phase[35,36]. So far, the retrograde memory deficits stem primarily from an impairment of consolidation processes; that is to say, to an inability to store new events over time, thereby reflecting an anterograde rather than a pure retrograde memory deficit. Thus, the question emerges to know whether retrograde memory impairments induced by benzodiazepines are not solely due to a weakness of the consolidation phase, but could also reflect a “pure” deficit of retrieval processes and in which conditions.

Paradoxically, retrieval processes have been implicated as a potential mechanism by which benzodiazepines can produce retrograde memory facilitation. Indeed, under certain circumstances, retrieval of information acquired before the benzodiazepine administration is not only spared, but even improved. It has been hypothesized that this phenomenon is not a true facilitation of retrieval processes, but is the result of reduced interference from items presented after drug administration and is thus a secondary consequence of drug-induced amnesia[37]. This hypothesis has been ruled out, nevertheless, by a further study demonstrating that at a given dose, lorazepam improved retrieval of events delivered prior to the benzodiazepine administration, in the absence of amnesia for the events presented after, thus excluding an interference explanation[38]. Interestingly, retrograde facilitation has also been observed as a result of an enhancement of automatic retrieval processes[39]. Animal experiments in rats also evidenced an enhancement of retrieval memory processes by benzodiazepines in a fear-conditioning paradigm[40] or in an active avoidance task[41].

Pure retrieval deficits have also been observed both in human and animals. Indeed, Pompeia et al.[42] have shown in humans that the benzodiazepine-induced impairment of memory depends more on the cues given at the retrieval than the retrieval instructions given to the subjects. Other studies performed in humans have also evidenced retrieval memory deficits[43,44]. Interestingly, retrieval memory deficits have also been found in rodents[45]. More specifically, we found that a pretest injection of diazepam induced forgetting for previously acquired spatial information in a delayed alternation task run in a T-maze; this diazepam-induced amnesia was totally reversed by adding a cardboard (without informative value) in the maze at the time of testing, as compared to noncued diazepam-treated animals[46]. This procedure, which avoided any state-dependent effect, demonstrated that diazepam produced a pure retrieval memory impairment. Interestingly, the pretest administration of methylbetacarboline (an inverse agonist of the benzodiazepine receptors) at a dose having anxiogenic properties produced an enhancement of retrieval processes in the same delayed alternation task[47]. Thus, these two studies suggested that the emotional state of the subject at the time of testing might be a key factor in the modulatory action of benzodiazepines at the time of retrieval.

The relationships between retrieval memory impairments and the emotional effects of benzodiazepines remain an open question; on the one hand, some studies showed a dissociation between the anxiolytic and the amnestic effects of benzodiazepines[3,48,49] and on the other hand, as stated by Eysenck[50], a moderate level of anxiety may benefit cognitive performance, depending on task difficulty. In agreement with this idea, we found that the amnestic effect of diazepam or, inversely, the promnesiant effect of β-carbolines in mice was directly linked to both the memory load of the task and to the anxiolytic/anxiogenic effects of the compounds[8,51]. From the very few studies evidencing a pure retrieval impairment in animals, it seems that retrieval deficits are more likely to be observed in subjects receiving a dose that has an effective emotional impact at the time of recall and submitted to a task involving an episodic-like memory component; the task difficulty, which induced different levels of searching strategies at the time of retrieval, might be also of critical importance in evidencing an interaction between the emotional impact of benzodiazepines and retrieval memory impairments[34,52]. This conclusion is in agreement with clinical studies that show that the frequency of conscious recollection for both true and false autobiographical memories in lorazepam-treated subjects is influenced by the emotion experienced at the time of retrieval[53].

## CONCLUSION

As emphasized above, the cognitive effects of benzodiazepines are not limited to explicit memory and to encoding processes. The possibility to dissociate different effects of several classes of ligands of the benzodiazepine receptors on memory types and memory stages provides an additional method for exploring the interaction and neural substrates of these different forms of memory. To this view, the study of the different impacts of benzodiazepines on retrieval processes in animals, whether based on implicit vs. explicit strategy, emerges as a key tool.

## ACKNOWLEDGMENTS

This study was supported by the CNRS. The author thanks Frances Ash (ashberac@free.fr) for translation.
